# A Case of High-Risk Acute Stanford Type B Aortic Dissection in a Right-Sided Aortic Arch Presenting With Transient Hemiparesis

**DOI:** 10.7759/cureus.114156

**Published:** 2026-08-08

**Authors:** Aoumar G Chamma, Wendy Saliba, Linda Chamma

**Affiliations:** 1 Cardiology, University of Balamand, Beirut, LBN; 2 Urology, Lebanese University, Beirut, LBN

**Keywords:** carotid-subclavian bypass, mirror-image branching, right-sided aortic arch, spinal cord protection, stanford type b aortic dissection, thoracic endovascular aortic repair, transient hemiparesis

## Abstract

Acute aortic dissection usually presents with abrupt chest or back pain, but neurological symptoms may dominate the presentation and mimic acute ischemic stroke. We report a case of a 50-year-old woman who presented with recurrent transient left-sided weakness. The initial episode persisted during early hospital assessment and resolved as brain magnetic resonance imaging was initiated. A second episode occurred during subsequent aortic computed tomography angiography and lasted approximately 20 minutes. Brain computed tomography and diffusion-weighted magnetic resonance imaging showed no acute abnormality, while magnetic resonance angiography of the head and neck showed no large-vessel occlusion or cervical arterial involvement.

A marked inter-arm blood pressure difference prompted computed tomography angiography of the chest, abdomen, and pelvis, which demonstrated an acute Stanford type B aortic dissection extending from the proximal descending thoracic aorta to the infrarenal abdominal aorta. The dissection occurred in a Type I right-sided aortic arch with mirror-image branching, consisting of a left brachiocephalic trunk followed by the right common carotid and right subclavian arteries. The dissected descending thoracic aorta measured up to 6.7 × 5.4 cm. The patent false lumen supplied the right renal artery, with delayed right renal enhancement suggesting impaired perfusion despite preserved serum creatinine.

These high-risk imaging findings supported early intervention after multidisciplinary evaluation. On hospital day 3, the patient underwent a single-session hybrid repair consisting of bilateral carotid-subclavian bypasses, proximal embolization of both subclavian arteries, and thoracic endovascular aortic repair using two overlapping stent grafts. Completion angiography showed exclusion of antegrade false-lumen flow through the treated proximal entry region and preserved right renal perfusion through distal false-lumen re-entry. Cerebrospinal fluid drainage and perioperative pressure augmentation were used for spinal cord protection.

The patient was discharged without neurological deficit or major postoperative complication. Clinical evaluation and surveillance computed tomography angiography were planned at the one-month post-repair interval. Completion angiography documented immediate procedural success, while durable technical success remained to be confirmed by surveillance computed tomography angiography because of the residual focal graft malapposition and continued false-lumen-dependent right renal perfusion. This case highlights the importance of considering acute aortic disease in patients with recurrent focal neurological deficits, normal cerebral imaging, and marked inter-arm blood pressure asymmetry, even in the absence of typical thoracic pain. It also illustrates how Type I right-sided aortic arch anatomy and severe arch angulation can complicate proximal landing-zone planning and require an individualized hybrid repair strategy.

## Introduction

Acute aortic syndrome includes aortic dissection, intramural hematoma, and penetrating aortic ulcer. These conditions may rapidly result in rupture or compromise perfusion of the brain, spinal cord, abdominal organs, or extremities [1-3].

Aortic dissection is classified according to the anatomical segments involved. Stanford type A dissection involves the ascending aorta, regardless of the location of the primary intimal tear. Stanford type B dissection does not involve the ascending aorta and usually begins within the descending thoracic aorta. In the DeBakey classification, type III dissection originates in the descending thoracic aorta and may remain confined to the thorax or extend into the abdominal aorta. This distinction guides treatment. Type A dissection usually requires emergency open surgical repair. Uncomplicated type B dissection is generally managed initially with anti-impulse medical therapy. Endovascular intervention is indicated when rupture or malperfusion syndrome is present and may be considered in selected stable patients with high-risk clinical or imaging features [1-4].

Abrupt severe chest, interscapular, or back pain is the classic presentation. Some patients experience only mild, atypical, or no pain, allowing neurological manifestations to become the principal reason for seeking medical attention [5-8]. Reported neurological manifestations include cerebral infarction, transient ischemic attack, syncope, encephalopathy, spinal cord ischemia, and peripheral ischemic neuropathy [7]. These atypical presentations may delay diagnosis and expose patients to thrombolytic or antithrombotic treatment before the underlying aortic emergency is recognized [7,8].

A right-sided aortic arch adds anatomical and technical complexity. Right-sided arches may be classified according to their supra-aortic branching pattern. A Type I right-sided aortic arch demonstrates mirror-image branching. From proximal to distal, the arch gives rise to a left brachiocephalic trunk, the right common carotid artery, and the right subclavian artery. The left brachiocephalic trunk subsequently divides into the left common carotid and left subclavian arteries [9]. This arrangement mirrors the usual branching pattern of a left-sided aortic arch. The branch configuration, together with the steep angulation that may accompany a right-sided arch, can affect cerebral and upper-extremity perfusion, wire and catheter behavior, stent-graft delivery, and the availability of an adequate proximal landing zone for thoracic endovascular aortic repair.

We describe a case of a 50-year-old woman who presented with recurrent transient hemiparesis and normal cerebral imaging. Further evaluation revealed a high-risk acute Stanford type B aortic dissection in a severely angulated Type I right-sided aortic arch with mirror-image branching. The dissection was associated with a patent false lumen, impaired right renal perfusion, and marked enlargement of the dissected descending thoracic aorta. Definitive management required bilateral carotid-subclavian revascularization, proximal embolization of both subclavian arteries, and thoracic endovascular aortic repair during a single hybrid operative session.

## Case presentation

A 50-year-old woman presented to the emergency department (hospital day 1) with sudden weakness of the left arm and leg. The symptoms had begun approximately 60 minutes before arrival and persisted during the initial hospital assessment. The National Institutes of Health Stroke Scale score during the initial deficit was 7, comprising 3 points for left-arm motor weakness, 3 points for left-leg motor weakness, and 1 point for level of consciousness, as she required minor stimulation to remain alert. She had no facial weakness, dysarthria, aphasia, sensory loss, visual disturbance, seizure, or complete loss of consciousness. She was a lifelong non-smoker and had no known history of hypertension, diabetes mellitus, dyslipidemia, coronary artery disease, previous stroke, connective tissue disease, or aortic disease. She reported mild epigastric discomfort but denied chest pain, back or interscapular pain, generalized abdominal pain, dyspnea, diaphoresis, nausea, vomiting, syncope, or presyncope. There was no history of recent trauma, stimulant use, pregnancy, hormonal therapy, or unusual physical exertion before presentation.

On arrival, her blood pressure was 170/80 mmHg in the right arm and 80/70 mmHg in the left arm. Her heart rate was 127 beats per minute, respiratory rate was 18 breaths per minute, oxygen saturation was 98% on room air, and temperature was 37.3°C.

The carotid, radial, femoral, and pedal pulses were palpable despite the pronounced inter-arm blood pressure difference. There were no clinical signs of upper or lower extremity ischemia. Cardiac auscultation revealed no new murmur or aortic regurgitation murmur.

A non-contrast computed tomography scan of the brain was performed approximately 15 minutes after arrival, corresponding to approximately 75 minutes after symptom onset. It showed no intracranial hemorrhage, established infarction, mass effect, or other acute abnormality. Brain magnetic resonance imaging, including diffusion-weighted imaging, and magnetic resonance angiography of the head and neck were initiated approximately 25 minutes after arrival, corresponding to approximately 85 minutes after symptom onset. The initial neurological deficit resolved while magnetic resonance imaging was being initiated. Diffusion-weighted magnetic resonance imaging showed no acute ischemic lesion, and no intracranial hemorrhage was identified. Magnetic resonance angiography showed no intracranial large-vessel occlusion requiring mechanical thrombectomy. It showed no cervical arterial occlusion or dissection involving the carotid or vertebral arteries.

After completion of the cerebral magnetic resonance studies, the patient proceeded to computed tomography angiography of the chest, abdomen, and pelvis. During this subsequent aortic imaging evaluation, the neurological deficit recurred for approximately 20 minutes. The recurrent episode reproduced the left arm and leg weakness and mild reduction in alertness, with the same National Institutes of Health Stroke Scale score of 7. It was followed by complete neurological recovery.

The stroke team was consulted. Intravenous thrombolysis was not administered because the combination of recurrent neurological deficits, mild epigastric discomfort, marked tachycardia, and an extreme inter-arm blood pressure difference raised concern for an alternative vascular diagnosis.

Aspirin, clopidogrel, heparin, and other antithrombotic agents were withheld during the initial diagnostic evaluation. Her high-sensitivity cardiac troponin I level was 3 pg/mL, below the laboratory upper reference limit of 14 pg/mL. The D-dimer concentration was 0.5 µg/mL fibrinogen-equivalent units, exactly at the assay cutoff and therefore not below the laboratory reference threshold of less than 0.5 µg/mL. Initial and subsequent laboratory findings are summarized in Table 1.

**Table 1:** Initial laboratory findings at presentation and subsequent laboratory investigations during hospitalization FEU: fibrinogen-equivalent units

Time point	Laboratory test	Result	Laboratory reference value
Presentation	Hemoglobin	12.8 g/dL	12-15 g/dL
Presentation	White blood cell count	10.8 × 10³/µL	4-11 × 10³ cells/µL
Presentation	Platelet count	192 × 10³/µL	150-450 × 10³/µL
Presentation	Serum creatinine	0.8 mg/dL	0.6-1.1 mg/dL
Presentation	Serum sodium	141 mmol/L	135-145 mmol/L
Presentation	Serum potassium	4.2 mmol/L	3.5-5.0 mmol/L
Presentation	C-reactive protein	3 mg/L	<10 mg/L
Presentation	High-sensitivity cardiac troponin I (hs-cTnI)	3 pg/mL	<14 pg/mL
Presentation	International normalized ratio	1.1	0.8-1.1
Presentation	D-dimer	0.5 µg/mL FEU	<0.5 µg/mL FEU
Before cerebrospinal fluid drain removal	Platelet count	210,000/µL	150,000-450,000/µL
Before cerebrospinal fluid drain removal	International normalized ratio	1.1	0.8-1.1
Before cerebrospinal fluid drain removal	Activated partial thromboplastin time	26 seconds	25-36 seconds
Postoperative period	Serum creatinine	0.8 mg/dL	0.6-1.1 mg/dL
Etiologic evaluation	Erythrocyte sedimentation rate	6 mm/hour	≤30 mm/hour
Etiologic evaluation	Antinuclear antibody test	Negative	Negative
Etiologic evaluation	Antineutrophil cytoplasmic antibody test	Negative	Negative
Etiologic evaluation	Heritable thoracic aortic disease multigene panel	No pathogenic or likely pathogenic variant detected	No pathogenic or likely pathogenic variant detected

Contrast-enhanced computed tomography angiography of the chest, abdomen, and pelvis was performed approximately 55 minutes after arrival, corresponding to approximately 115 minutes after initial symptom onset. The ascending aorta measured 3.8 cm at the level of the main pulmonary artery. The aortic arch was ectatic, right-sided, and severely angulated. Computed tomography angiography demonstrated a Type I right-sided aortic arch with mirror-image branching. The first branch was a left brachiocephalic trunk, which divided into the left common carotid and left subclavian arteries. The second and third branches were the right common carotid and right subclavian arteries, respectively.

**Figure 1:**
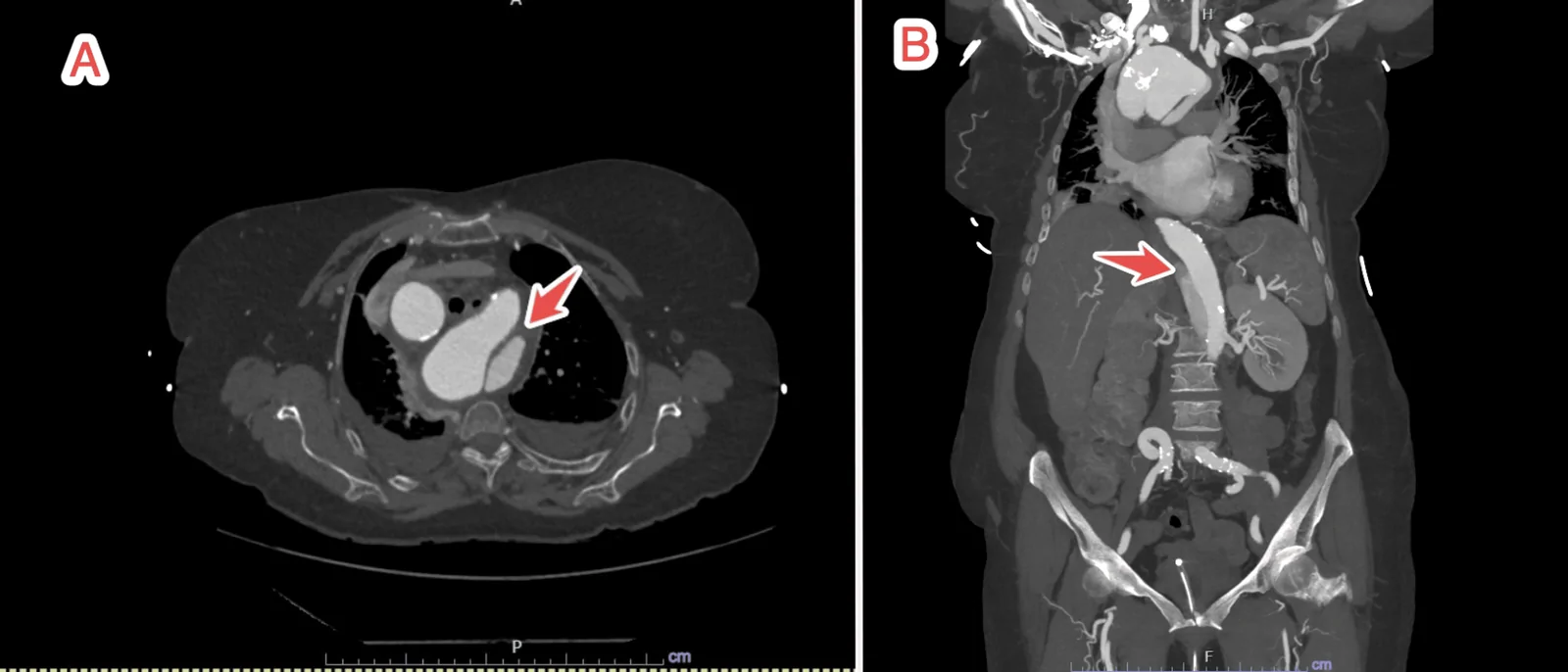
Preoperative computed tomography angiography showing acute Stanford type B aortic dissection. (A) Axial contrast-enhanced computed tomography image showing the dissected descending thoracic aorta with the intimal flap separating the true lumen from the patent false lumen. (B) Coronal reformatted image showing longitudinal extension of the dissection through the descending thoracic and abdominal aorta. The arrows indicate the dissection flap and patent false lumen.

The supra-aortic vessels remained patent, and no extension of the dissection into the cervical arteries was identified. The dissection began in the proximal descending thoracic aorta and extended through the descending thoracic and abdominal aorta to the infrarenal segment, without involvement of the ascending aorta (Figure 1).

The dissected descending thoracic aorta was markedly enlarged, measuring up to 6.7 × 5.4 cm. A crescent-shaped false lumen was present, while the true lumen measured up to 3.7 × 4.0 cm. The false lumen remained patent, as demonstrated by contrast opacification and communication with the right renal artery.

The celiac artery and superior mesenteric artery were patent and originated from the true lumen. The left renal artery also originated from the true lumen. The right renal artery remained patent but originated from the false lumen. Mildly delayed enhancement of the right kidney suggested impaired right renal perfusion despite preserved serum creatinine. Posterior mediastinal edema and inflammatory fat stranding were present, together with a trace right pleural effusion. There was no pericardial effusion, free intraperitoneal fluid, active contrast extravasation, or radiological evidence of rupture.

Computed tomography angiography showed high-risk features, including marked enlargement of the dissected descending thoracic aorta and imaging evidence of impaired right renal perfusion. The patient had no documented oliguria, renal failure, renal infarction, refractory hypertension, rupture, or other clinical evidence of established malperfusion syndrome. The dissection was therefore classified as high risk rather than definitively complicated. These findings supported early intervention after multidisciplinary evaluation. The recurrent neurological symptoms were temporally associated with the acute aortic presentation, but no direct anatomical mechanism was demonstrated.

Intravenous esmolol was started on hospital day 1 for anti-impulse therapy. The initial therapeutic targets were a heart rate of 60-80 beats per minute and a systolic blood pressure of 100-120 mmHg, provided adequate cerebral, renal, and peripheral perfusion was maintained. Morphine 2 mg was administered intravenously for analgesia.

Following hemodynamic stabilization and multidisciplinary evaluation by the vascular, endovascular, anesthesia, and critical care teams, hybrid repair was selected. Operative planning incorporated the Type I mirror-image branching pattern, severe arch angulation, the proximal dissection entry region, and the intended proximal stent-graft seal zone. The planned proximal coverage required protection of both vertebral and upper-extremity circulations before exclusion of the intended aortic segment. Bilateral carotid-subclavian bypasses and proximal embolization of both subclavian arteries were therefore planned before thoracic endovascular aortic repair. This strategy was intended to preserve bilateral upper-extremity and vertebral artery perfusion while reducing retrograde flow into the excluded aortic segment.

A lumbar cerebrospinal fluid drainage catheter was placed at the L3-L4 interspace as part of the institutional spinal cord protection protocol. Cerebrospinal fluid pressure was maintained at or below 10 mmHg using intermittent drainage, limited to 10 mL per hour and 150 mL over 24 hours according to the institutional protocol.

On hospital day 3, all components of the repair were performed sequentially during a single hybrid operative session under general anesthesia. Bilateral carotid-subclavian revascularization and proximal subclavian embolization were completed first, followed by thoracic endovascular aortic repair. Mean arterial pressure was maintained between 90 and 100 mmHg during thoracic endovascular aortic repair and throughout the immediate postoperative period to support spinal cord perfusion. Systemic heparinization was initiated with 5,000 IU of unfractionated heparin and subsequently guided by intraoperative activated clotting time monitoring. An 8-mm Dacron graft was used for each carotid-subclavian bypass.

The right subclavian artery was accessed under fluoroscopic guidance, and angiography was performed to identify the right vertebral artery origin. Deployment of a 16 × 12 mm Amplatzer Vascular Plug II (Abbott, Illinois, USA) was unsuccessful because of arterial tortuosity and excessive device oversizing. A smaller 12 × 9 mm Amplatzer Vascular Plug II was then successfully deployed in the prevertebral segment of the right subclavian artery. Angiography confirmed proximal right subclavian occlusion with preservation of vertebral artery perfusion. The right carotid-subclavian bypass was completed, with restoration of a strong right radial pulse. The left subclavian artery was subsequently accessed, and a 14 × 10 mm Amplatzer Vascular Plug II was deployed in the prevertebral segment. Completion angiography demonstrated satisfactory proximal occlusion with preservation of the left vertebral artery origin.

**Figure 2:**
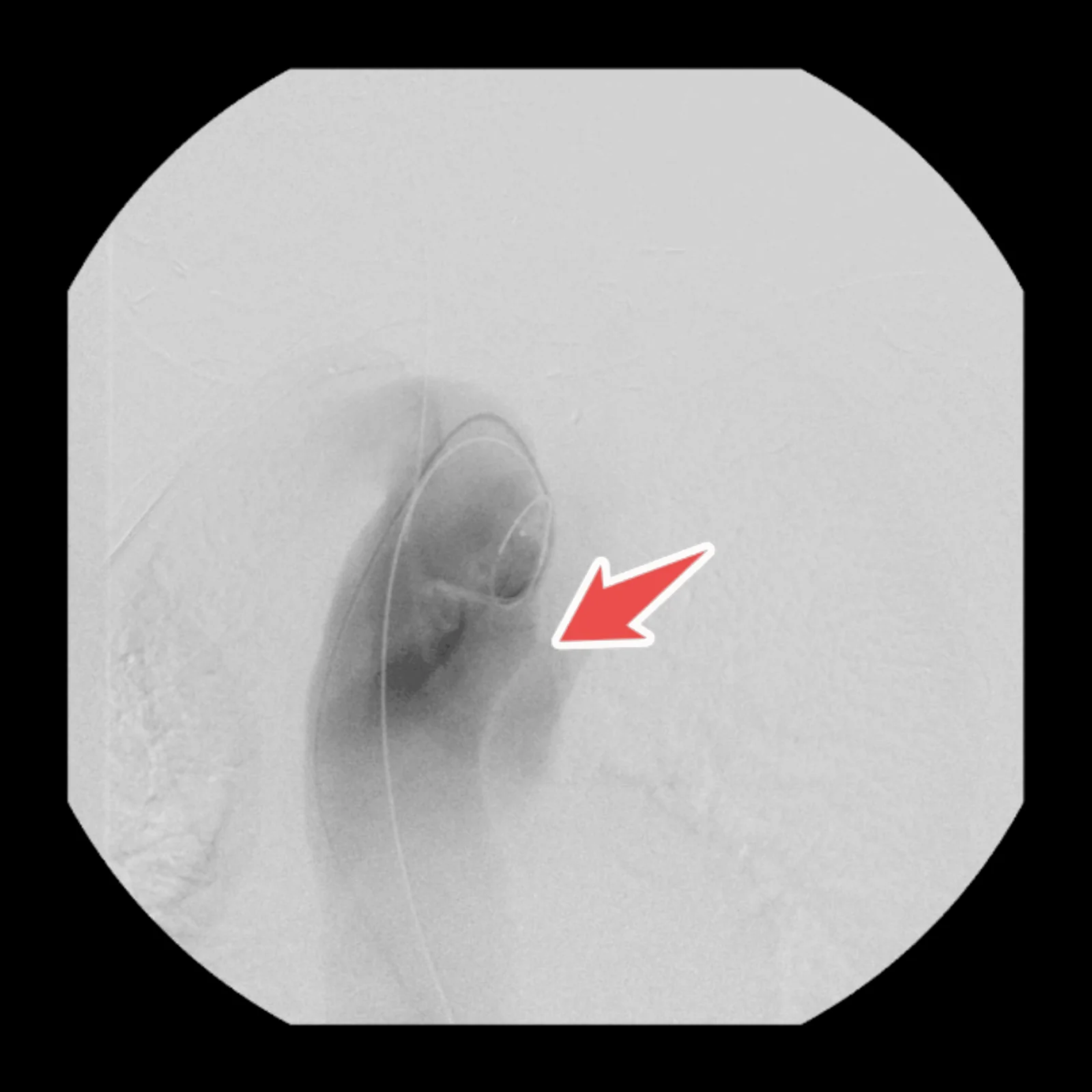
Pre-deployment angiography demonstrating the thoracic aortic dissection (arrow) before thoracic endovascular aortic repair

An 8-mm left carotid-subclavian bypass was then completed, with a strong left radial pulse following reperfusion. After completion of both bypasses and subclavian embolizations, bilateral femoral arterial access was obtained. A guidewire and catheter were advanced through the aortic true lumen. Pre-deployment aortography confirmed true-lumen catheterization and demonstrated the dissected thoracic aorta before thoracic endovascular aortic repair (Figure 2).

The celiac artery, superior mesenteric artery, and left renal artery originated from the true lumen, while the right renal artery continued to originate from the false lumen. A conformable thoracic aortic stent graft measuring 37 × 37 mm in diameter and 20 cm in length was deployed immediately distal to the right common carotid artery. Proximal positioning was technically difficult because of the severe angulation of the right-sided aortic arch. A second overlapping stent graft measuring 37 × 37 mm and 15 cm in length was implanted to extend distal thoracic coverage and provide adequate overlap between the devices. The exact overlap length was not documented in the available operative report, so the effective total aortic coverage length could not be calculated.

**Figure 3:**
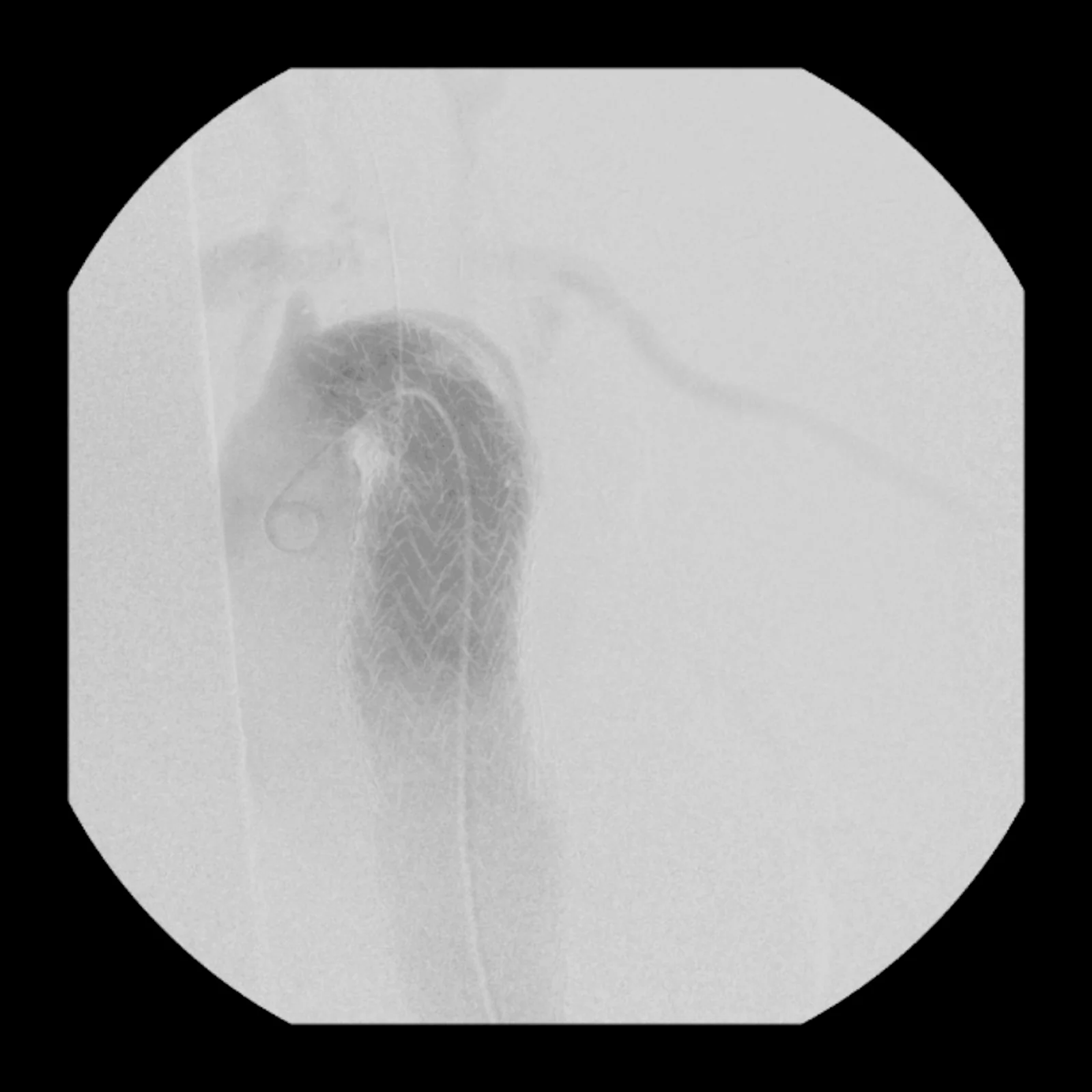
Completion angiography showing the stent-graft position following thoracic endovascular aortic repair

The proximal graft was subsequently molded using a Tri-Lobe balloon to improve proximal apposition. Completion angiography after thoracic endovascular aortic repair showed exclusion of antegrade false-lumen flow through the treated proximal entry region (Figure 3).

The celiac artery, superior mesenteric artery, and left renal artery remained perfused through the true lumen. Preserved right renal perfusion was demonstrated through distal false-lumen re-entry. Both carotid-subclavian bypasses remained patent, with preserved vertebral and upper-extremity perfusion. A small area of incomplete stent-graft apposition remained along the greater curvature of the aortic arch. Despite this focal malapposition, the treated proximal entry region appeared sealed on completion angiography.

Bilateral radial and femoral pulses were present at the end of the procedure, and no intraoperative complication was reported. The patient was transferred intubated to the intensive care unit following the hybrid repair. Mean arterial pressure was maintained between 90 and 100 mmHg during the immediate postoperative period. She was extubated on the first postoperative day and remained in the intensive care unit for two days.

The cerebrospinal fluid drainage catheter was maintained for 24 hours after thoracic endovascular aortic repair and clamped for a further 24 hours during serial neurological assessment. Before catheter removal, the platelet count was 210,000/µL, the international normalized ratio was 1.1, and the activated partial thromboplastin time was 26 seconds, all within their respective laboratory reference ranges (Table 1). The catheter was removed on postoperative day 2 without complication, following which aspirin 81 mg once daily was initiated. Aspirin monotherapy was selected over dual antiplatelet therapy after balancing the increased bleeding risk associated with recent lumbar cerebrospinal fluid drainage and extensive mediastinal dissection against the need for antiplatelet protection of the bilateral prosthetic carotid-subclavian bypass grafts.

Serial neurological examinations showed no recurrent hemiparesis, stroke, paraplegia, paraparesis, sensory level, or other evidence of spinal cord ischemia. The patient had no upper extremity ischemia, and bilateral radial pulses remained palpable. There was no post-dural puncture headache, cerebrospinal fluid leakage, bleeding, infection, catheter malfunction, intracranial or spinal hematoma, or drain-related neurological deficit.

Renal function remained stable, with a postoperative serum creatinine level of 0.8 mg/dL (Table 1) and no clinical evidence of progressive renal malperfusion. No postoperative bleeding, access-site complication, respiratory complication, myocardial ischemia, or clinically apparent graft-related complication was identified.

She was discharged on hospital day 6, corresponding to postoperative day 3, without residual neurological deficit and with a modified Rankin Scale score of 0. Her discharge medications were bisoprolol 5 mg orally once daily, losartan 50 mg orally once daily, and aspirin 81 mg orally once daily. She was advised to maintain strict long-term blood pressure control and avoid heavy isometric exercise or activities associated with abrupt increases in arterial pressure.

Clinical evaluation and surveillance computed tomography angiography were planned at the one-month post-repair interval. At the time of manuscript preparation, the one-month follow-up date had not yet been reached. The planned computed tomography angiography was intended to assess stent-graft position and apposition, endoleak, false-lumen thrombosis and remodeling, right renal perfusion, and the patency of both carotid-subclavian bypasses.

Because of her age at presentation and the absence of established cardiovascular risk factors, the patient underwent genetic counseling and testing for heritable thoracic aortic disease. She reported no family history of aortic aneurysm, aortic dissection, unexplained sudden death, or premature or unexplained stroke. Physical examination showed no findings suggestive of Marfan syndrome. She had no tall or disproportionate stature, pectus deformity, scoliosis, positive wrist or thumb signs, or history or clinical evidence of lens dislocation. She had no features suggestive of Loeys-Dietz syndrome, including hypertelorism, bifid uvula, or clinically apparent generalized arterial tortuosity. No findings suggestive of vascular Ehlers-Danlos syndrome were present, including translucent skin, unexplained easy bruising, or marked joint hypermobility.

Transthoracic echocardiography showed a tricuspid aortic valve without dilatation of the aortic root or ascending aorta. A targeted heritable thoracic aortic disease multigene panel, including FBN1, TGFBR1, TGFBR2, SMAD3, TGFB2, COL3A1, ACTA2, MYH11, MYLK, PRKG1, and LOX, identified no pathogenic or likely pathogenic variants. The negative result reduced the likelihood of a currently recognized monogenic aortopathy but did not completely exclude a heritable predisposition. The erythrocyte sedimentation rate was 6 mm/hour, below the laboratory reference limit of 30 mm/hour, while antinuclear and antineutrophil cytoplasmic antibody tests were negative (Table 1).

No clinical, laboratory, or imaging findings strongly suggested an inflammatory aortopathy. Screening aortic imaging was recommended for her first-degree relatives despite the negative genetic panel.

## Discussion

This case is notable for the presentation of high-risk acute Stanford type B aortic dissection with recurrent transient neurological symptoms, absence of typical severe thoracic pain, and the technical complexity created by a severely angulated Type I right-sided aortic arch with mirror-image branching. Neurological complications are well recognized in acute aortic syndromes; they occur more commonly and are anatomically easier to explain in type A dissection because the ascending aorta and arch vessels are directly involved [1,4,7]. Transient unilateral cerebral symptoms are more difficult to attribute directly to a type B dissection that begins distal to the arch when the supra-aortic arteries remain patent.

In this patient, computed tomography angiography showed no extension of the dissection into the carotid or vertebral arteries and no fixed obstruction of the supra-aortic vessels. Magnetic resonance angiography of the head and neck showed no large-vessel occlusion or cervical arterial dissection, and brain diffusion-weighted magnetic resonance imaging showed no acute ischemic lesion. The mechanism of the transient hemiparesis therefore remained uncertain. A brief hemodynamic disturbance involving the complex arch circulation or a transient embolic event before vascular imaging was possible. A coincidental transient ischemic attack could not be excluded. This cautious interpretation is important because dynamic true-lumen compression in type B dissection more readily explains renal, mesenteric, spinal, or limb malperfusion than unilateral cerebral symptoms when the dissection does not involve the arch vessels. Similarly, a direct embolic pathway from the descending false lumen to the cerebral circulation was not demonstrated. The close temporal relationship between the neurological episodes and the acute aortic presentation supports a possible association but does not establish causality.

Normal diffusion-weighted magnetic resonance imaging does not exclude a transient ischemic event. Brief hemodynamic or embolic disturbances may resolve without producing permanent tissue injury detectable by diffusion imaging [7]. Complete recovery after both episodes and the absence of an acute cerebral lesion supported a transient rather than completed ischemic process.

The absence of severe chest or back pain created an important diagnostic challenge. Painless or minimally painful dissections are more likely to be overlooked, particularly when focal neurological deficits dominate the presentation [5,6,8]. In this patient, mild epigastric discomfort, tachycardia, and the extreme inter-arm blood pressure difference were the principal clues that the presentation might not represent an isolated transient ischemic attack. The inter-arm blood pressure difference was particularly important because all peripheral pulses remained palpable. Pulse abnormalities in acute dissection may be intermittent, incomplete, or masked by collateral circulation. Bilateral blood pressure measurement may therefore reveal clinically significant perfusion asymmetry even when the pulse examination appears reassuring [1,5].

The Type I mirror-image branching pattern was identified on computed tomography angiography. However, no fixed stenosis, occlusion, or dissection involving the left brachiocephalic trunk, left subclavian artery, or other supra-aortic branches was demonstrated. Transient or dynamic impairment of branch-vessel flow remains a possible explanation for the marked left-arm hypotension, but the precise mechanism could not be established.

Acute ischemic stroke and aortic dissection require markedly different immediate treatments. Thrombolysis in an unrecognized aortic dissection may increase the risk of rupture, dissection extension, hemopericardium, and major internal bleeding [1,7,8]. In this patient, the marked inter-arm pressure difference and atypical pain prompted investigation for an alternative vascular diagnosis before thrombolytic or antithrombotic therapy was administered.

The D-dimer value at the laboratory cutoff also demonstrates why biomarkers should not override clinical probability. D-dimer has good sensitivity but limited specificity, and its diagnostic performance varies with the assay, symptom duration, anatomical extent, and false-lumen characteristics [10,11]. Aortic dissection detection risk score and D-dimer pathways are most useful in carefully selected patients with low or intermediate clinical probability. They should not be used to exclude acute aortic syndrome when major warning signs are present [12,13].

Computed tomography angiography showed marked enlargement of the dissected descending thoracic aorta to 6.7 cm and delayed enhancement of the right kidney, which was supplied by the patent false lumen. Delayed renal enhancement may precede an increase in serum creatinine, so preserved renal function does not exclude impaired renal perfusion. Malperfusion can result from static branch-vessel involvement, dynamic compression of the true lumen, or a combination of both mechanisms [1-4].

The available imaging established false-lumen supply of the right renal artery but did not fully define the relative contributions of static and dynamic obstruction. The patient did not have documented renal failure, oliguria, renal infarction, rupture, or another clinical manifestation of established malperfusion syndrome. The most defensible classification was therefore high-risk type B aortic dissection with imaging evidence of impaired right renal perfusion rather than definitively complicated dissection.

Marked enlargement of the dissected descending thoracic aorta and impaired right renal enhancement supported early intervention after multidisciplinary evaluation. Thoracic endovascular aortic repair is indicated in anatomically suitable patients with complicated type B dissection and may be considered in selected stable patients with high-risk features when the anticipated benefits outweigh the procedural risks [2-4,14].

Closure of the proximal entry region can reduce false-lumen pressurization, expand the true lumen, improve branch-vessel perfusion when dynamic obstruction is present, and promote favorable aortic remodeling. The patient’s arch anomaly was classified as a Type I right-sided aortic arch with mirror-image branching. In this configuration, the first branch is the left brachiocephalic trunk, followed by the right common carotid and right subclavian arteries [9]. The left brachiocephalic trunk supplies the left common carotid and left subclavian arteries. Correct identification of this pattern is important because it differs from a Type II right-sided aortic arch with an aberrant left subclavian artery. These configurations produce different relationships between the supra-aortic vessels, descending aorta, and potential proximal stent-graft seal zones. In this patient, severe arch angulation and the relationship between the supra-aortic branches, vertebral arteries, proximal dissection entry region, and intended seal zone made thoracic endovascular aortic repair without prior branch-vessel protection unsuitable.

Bilateral carotid-subclavian bypasses maintained bilateral upper extremity and vertebral artery perfusion. Proximal subclavian embolization was used to reduce retrograde flow into the excluded aortic segment. The bypass and embolization procedures were completed before stent-graft deployment so that upper extremity and posterior cerebral circulations were protected before endovascular exclusion of the intended proximal segment.

Completion angiography showed continued right renal perfusion through distal false-lumen re-entry after exclusion of antegrade flow through the treated proximal entry region. This finding was important because the right renal artery originated from the false lumen. Complete thrombosis of the entire false lumen was neither demonstrated nor expected immediately after proximal exclusion in the presence of distal re-entry channels. Follow-up cross-sectional imaging remained necessary to assess renal perfusion and subsequent false-lumen remodeling.

The anticipated extensive thoracic aortic coverage placed the patient at increased risk of spinal cord ischemia. A multimodal protection strategy was therefore used, combining selective cerebrospinal fluid drainage, preservation of both subclavian circulations, perioperative pressure augmentation, and frequent neurological assessment. These measures were intended to maintain spinal cord perfusion by supporting systemic arterial pressure while limiting cerebrospinal fluid pressure, and no spinal cord deficit occurred [4,15].

Focal incomplete graft apposition may increase the risk of proximal endoleak, device migration, or later graft-related complications. Because the malapposition was described along the greater curvature, it could not be classified as a conventional bird-beak configuration without postoperative cross-sectional imaging. A conventional bird-beak configuration is generally defined by a wedge-shaped proximal gap along the inner or lesser curvature of the arch [16]. The absence of visible antegrade false-lumen flow through the treated proximal entry region was reassuring, but the residual focal malapposition strengthened the need for early imaging surveillance.

The postoperative antiplatelet regimen required a balance between bleeding and thrombotic risks. Recent lumbar cerebrospinal fluid drainage and extensive hybrid surgical dissection increased concern regarding early bleeding, while the bilateral prosthetic carotid-subclavian bypass grafts required antiplatelet protection. Aspirin monotherapy was therefore selected instead of dual antiplatelet therapy. This decision was consistent with retrospective evidence showing a lower incidence of bleeding with single antiplatelet therapy after left subclavian artery revascularization during thoracic endovascular aortic repair, without a significant increase in ischemic events during six months of follow-up [17].

Because the dissection occurred before 60 years of age without established cardiovascular risk factors, the patient underwent evaluation for heritable thoracic aortic disease. The negative multigene panel reduced the likelihood of a recognized monogenic disorder but did not exclude an unidentified genetic predisposition. Screening aortic imaging was therefore recommended for first-degree relatives [1,18].

Long-term management after repair should focus on lifelong anti-impulse therapy, strict blood pressure control, and serial aortic imaging. The patient was discharged on bisoprolol and losartan to reduce heart rate, arterial pressure, and aortic wall stress. Clinical evaluation and surveillance computed tomography angiography were planned at the one-month post-repair interval because of the residual dissected aorta, false-lumen-dependent right renal artery, and focal proximal graft malapposition. Subsequent imaging at 6 and 12 months, and annually thereafter, would depend on the early findings and subsequent aortic stability [1].

## Conclusions

Recurrent focal neurological deficits with normal cerebral imaging should prompt consideration of an aortic or systemic vascular cause when accompanied by marked inter-arm blood pressure asymmetry or atypical thoracoabdominal symptoms. In this patient, the bedside pressure difference led to the diagnosis of high-risk acute Stanford type B aortic dissection before thrombolytic or antithrombotic therapy was administered, although the precise relationship between the dissection and transient hemiparesis remained uncertain. Type I right-sided aortic arch with mirror-image branching must be defined precisely because its branch order, vessel geometry, and associated arch angulation can substantially affect endovascular access, proximal seal-zone planning, and the need for supra-aortic revascularization. An individualized single-session hybrid repair achieved immediate exclusion of antegrade flow through the treated proximal entry region while preserving vertebral, upper extremity, visceral, and renal perfusion on completion angiography. Durable technical success remained to be confirmed by the planned surveillance computed tomography angiography. Successful repair does not eliminate future aortic risk, and lifelong blood pressure control, family screening, and continued cross-sectional surveillance remain essential.
